# Mouse Models for Pseudoxanthoma Elasticum: Genetic and Dietary Modulation of the Ectopic Mineralization Phenotypes

**DOI:** 10.1371/journal.pone.0089268

**Published:** 2014-02-19

**Authors:** Qiaoli Li, Haitao Guo, David W. Chou, Annerose Berndt, John P. Sundberg, Jouni Uitto

**Affiliations:** 1 Department of Dermatology and Cutaneous Biology, Thomas Jefferson University, Philadelphia, Pennsylvania, United States of America; 2 Department of Medicine, University of Pittsburgh, Pittsburgh, Pennsylvania, United States of America; 3 The Jackson Laboratory, Bar Harbor, Maine, United States of America; Osaka University Graduate School of Medicine, Japan

## Abstract

Pseudoxanthoma elasticum (PXE), a heritable ectopic mineralization disorder, is caused by mutations in the *ABCC6* gene. Null mice (*Abcc6^−/−^*) recapitulate the genetic, histopathologic and ultrastructural features of PXE, and they demonstrate early and progressive mineralization of vibrissae dermal sheath, which serves as a biomarker of the overall mineralization process. Recently, as part of a mouse aging study at The Jackson Laboratory, 31 inbred mouse strains were necropsied, and two of them, KK/HlJ and 129S1/SvImJ, were noted to have vibrissae dermal mineralization similar to *Abcc6^−/−^* mice. These two strains were shown to harbor a single nucleotide polymorphism (rs32756904) in the *Abcc6* gene, which resulted in out-of-frame splicing and marked reduction in ABCC6 protein expression in the liver of these mice. The same polymorphism is present in two additional mouse strains, DBA/2J and C3H/HeJ, with similar reduction in Abcc6 protein levels, yet these mice did not demonstrate tissue mineralization when kept on standard rodent diet. However, all four mouse strains, when placed on experimental diet enriched in phosphate and low in magnesium, developed extensive ectopic mineralization. These results indicate that the genetic background of mice and the mineral composition of their diet can profoundly modulate the ectopic mineralization process predicated on mutations in the *Abcc6* gene. These mice provide novel model systems to study the pathomechanisms and the reasons for strain background on phenotypic variability of PXE.

## Introduction

Pseudoxanthoma elasticum in humans (PXE, OMIM#264800), an autosomal recessive Mendelian disorder, is characterized by multisystem ectopic mineralization with clinical manifestations primarily in the skin, the eyes, and the cardiovascular system [1], [2]. The clinical manifestations are of late onset, yet progressive, leading to significant morbidity and occasional mortality. PXE is caused in most cases by mutations in the *ABCC6* gene, which encodes a putative transmembrane efflux transporter primarily expressed in the liver and to a lesser extent in the kidneys. However, ABCC6 is found at very low levels, if at all, in clinically affected tissues [3].

Understanding of the pathophysiological mechanisms of PXE has been greatly advanced by development of animal models, particularly null mice through targeted ablation of the mouse *Abcc6* gene [4], [5]. These mutant mice recapitulate the genetic, histopathological, and ultrastructural features encountered in patients with PXE. A characteristic finding in the targeted mutant mice is mineralization of the vibrissae dermal sheath, a connective tissue capsule surrounding the dermal bulb of vibrissae. This mineralization develops in *Abcc6^−/−^* mice as early as 5–6 weeks of age and progresses with advancing age when the animals are fed normal laboratory diet [5], [6]. Thus, vibrissae mineralization serves as an early biomarker of the mineralization process and reflects the overall involvement of the internal organs of these mice. The vibrissa hair follicle is a specialized structure not found in humans, and mineralization of the vibrissae dermal sheath is unusual in mice. As part of a large scale mouse aging study, performed at The Jackson Laboratory, Bar Harbor, Maine, 31 inbred mouse strains were necropsied at 6, 12, and 20 months of age and at a moribund state [7]. Mineralization of vibrissae dermal sheaths, similar to that noted in *Abcc6^−/−^* mice, was diagnosed histologically in KK/HlJ mice at 6 months of age and older [8], [9]. Further histopathological examination of these mice revealed mineralization of internal organs similar to the *Abcc6* null mice, including medium-sized arteries, retina, lung, and dermis. Haplotype analysis of *Abcc6* revealed the presence of a non-synonymous single-nucleotide polymorphism (SNP) associated with tissue mineralization in KK/HlJ mice [8]. Specifically, a SNP in exon 14 (rs32756904) in the *Abcc6* gene displayed an A allele at base pair position 53,257,951 on chromosome 7 in KK/HlJ mice with vibrissae mineralization, and this SNP was subsequently shown to cause aberrant splicing of *Abcc6* pre-mRNA, resulting in an out-of-frame mRNA transcript. This polymorphism was previously noted, with similar consequences on *Abcc6* pre-mRNA splicing, also in C3H/HeJ mice [9], [10], and the presence of this polymorphism has been described in addition to C3H/HeJ in DBA/2J mice linked to dystrophic cardiac calcification (Dyscalc) phenotype [11]. This frame shift due to altered splicing results in a premature termination codon of translation and markedly reduced ABCC6 protein expression in the liver [9]. Initial histopathologic examination of these mice revealed some evidence of mineralization in the dermal sheath of vibrissae in 129S1/SvImJ mice, while no mineralization was noted in C3H/HeJ and DBA/2J mice, when kept on standard rodent diet [8] (Table 1).

**Table 1:** Vibrissae dermal sheath mineralization in different mouse strains examined in this study.

Strain	SNP rs32756904	On normal diet (≥20 mo)			On acceleration diet (5 mo)			
		No. of mice examined	Frequency (%)	Severity		No. of mice examined	Frequency (%)	Severity
KK/HlJ	A	23	78.3	++++		8	100.0	++++
DBA/2J	A	25	0.0	–		5	40.0	++
C3H/HeJ	A	34	0.0	–		7	57.1	++
129S1/SvImJ	A	41	22.0	+		10	90.0	+++
C57BL/6J	G	45	0.0	–		10	0.0	–
*Abcc6^−/−^*	G	13	100.0	++++		9	100.0	++++

The table shows the frequency and severity of vibrissae dermal sheath mineralization in different strains: −, not present; +, mild; ++++, severe. Mice were placed on either normal diet or acceleration diet at 4 weeks of age and tissues were collected for histopathology. The frequency values represent the percent of mice affected by vibrissae dermal sheath mineralization, as examined by Hematoxylin-Eosin stain on one section.

In this study, we compared the consequences of the mutations in all four inbred mouse strains depicting the frame shift mutation in the *Abcc6* gene at the mRNA and protein levels with respect to phenotypic changes of aberrant mineralization, as well as the effect of an experimental diet that accelerates mineralization in these mice.

## Materials and Methods

### Mice and Diet

The KK/HlJ, DBA/2J, C3H/HeJ, 129S1/SvImJ, and C57BL/6J mice were initially part of a large-scale aging study by The Jackson Aging Center, for which details have been described elsewhere [7]. Mice were maintained at the Breeding Facility of The Jackson Laboratory under standard conditions. Mice were allowed *ad libitum* access to acidified water (pH 2.8–3.2) and placed on rodent diet (LabDiet 5K52, PMI Nutritional International, Bentwood, MO). Euthanasia was performed by CO_2_ asphyxiation with methods approved by the American Veterinary Medical Association. All protocols were reviewed and approved by the Institutional Animal Care and Use Committee of The Jackson Laboratory. Proper handling and care were followed according to animal welfare policies of the Public Health Service.

The *Abcc6^tm1JfK^* mouse, a model for PXE, was developed by targeted ablation of the mouse *Abcc6* gene (hereafter referred to as *Abcc6^−/−^*) [5]. *Abcc6^−/−^* mice were made congenic onto the C57BL/6J background by 10 backcross generations. These mice, together with KK/HlJ (JR2106), DBA/2J (JR671), C3H/HeJ (JR659), 129S1/SvImJ (JR2448), and C57BL/6J (JR664) inbred (wild-type) mice, which were purchased from The Jackson Laboratory (Bar Harbor, ME), were housed in the Animal Facility of Thomas Jefferson University where they were maintained in a climate-controlled environment with free access to water and a 12-hours light/dark cycle. Mice were placed on either standard rodent diet (Laboratory Autoclavable Meal Rodent Diet 5010; PMI Nutrition, Brentwood, MO) or fed an “acceleration diet” (Harlan Teklad, Rodent diet TD.00442, Madison, WI), which is enriched in phosphorus and has reduced magnesium content [12]. This study was approved by the Institutional Animal Care and Use Committee of Thomas Jefferson University (approval no. 253F).

### Histological Analysis

After euthanization (CO_2_ asphyxiation) of mice, biopsies from muzzle skin containing vibrissae were collected and fixed in 10% phosphate-buffered formalin and embedded in paraffin. Tissues were sectioned (6 µm) and stained with hematoxylin and eosin (H&E) or Alizarin Red using standard procedures. Slides were examined under light microscopy for mineralization.

### RNA Extraction and Reverse Transcription Polymerase Chain Reaction

Total RNA from mouse liver was isolated with Trizol reagent (Invitrogen, Carlsbad, CA), followed by an RNeasy Mini Kit (Qiagen, Valencia, CA). The RNA was then treated in mini-columns with DNase I to eliminate genomic DNA. First-strand cDNA was synthesized with reverse transcriptase and random hexamer primers (Invitrogen) using 2 µg of RNA in each reaction. To amplify the regions flanking the 5-bp deletion in the *Abcc6* gene, we used a primer pair (5′-CGAGTGTCCTTTGACCGGCT-3′; 5′-TGGGCTCTCCTGGGACCAA-3′) that produces a 144-bp or 139-bp PCR fragment, corresponding to wild-type or mutant alleles, respectively, as visualized by high-resolution electrophoresis on a 15% MINI-PROTEAN Precast Gel (Bio-Rad, Hercules, CA) and ethidium bromide staining.

### Sanger Sequencing

Sequencing of PCR products from genomic and cDNA from different mouse strains was performed in the DNA Core Facility of the Kimmel Cancer Center at Thomas Jefferson University using an Applied Biosystems 3730 Sequencer (Applied Biosystems, Foster city, CA). Results were visualized with Chromas software (Technelysium, South Brisbane, Australia).

### ABCC6 Protein Sequence Alignments

The alignments of the ABCC6 protein sequences in different species were generated with the Clustal W program (http://www.ebi.ac.uk/tools/clustalw2).

### Real-time PCR

Real-time PCR of the mouse *Abcc6* gene was conducted using Power SYBR Green PCR Master Mix with the ABI Prism 7000 sequence detection system (Applied Biosystems), as described previously [9], 4–5 mice per group.

### Immunofluorescence

Livers from euthanized mice were quickly harvested and embedded in Optimal Cutting Temperature (OCT), and sectioned (6 µm) onto glass slides. Tissues were fixed in 4% paraformaldehyde before immunostaining with ABCC6-specific antibody S-20 (Santa Cruz Biotechnology, Inc., Santa Cruz, CA). Samples were then incubated at room temperature with Alexa Fluor 488 donkey anti-goat secondary antibody (Invitrogen), followed by DAPI staining of nuclei. Slides were viewed under a Carl Zeiss LSM 510 UV META inverted confocal microscope (Carl Zeiss Microimaging, Thornwood, NY) with a Plan-Apo 63×oil immersion lens using Zeiss AIM 4.2 SP1 software.

### Plasma Membrane Isolation and Western Blot

Plasma membranes from mouse liver were prepared as described previously [13]. A BCA kit (Thermo Scientific, Rockford, IL) was used to determine the concentration of plasma membrane proteins. After protein separation on 8% SDS-PAGE, an anti-ABCC6 antibody (S-20) made in goat, was used to detect the ABCC6 protein on a PVDF membrane. A mouse monoclonal anti-alpha 1 sodium potassium ATPase antibody was used as a plasma membrane marker for equal loading of proteins. To visualize the signal, the membrane was incubated with secondary antibodies (LI-COR, Lincoln, NE) and scanned with an Odyssey Infrared Imager (LI-COR). Results were analyzed in LI-COR ImageStudio Pro software.

### Statistical Analysis

The statistical significance between results in different groups of mice was evaluated by two-tailed Student’s t-test, and non-parametric Kruskal-Wallis test was conducted to test for global differences. The p-values ≤0.05 were considered statistically significant.

## Results

### Characterization of the *Abcc6* Mutant Mice

As reported previously, four different inbred strains of mice have been shown to harbor a specific SNP in the *Abcc6* gene, which in KK/HlJ mice, when kept on standard rodent diet, results in extensive aberrant mineralization, while the three other strains with the same polymorphism demonstrate very little, if any, mineralization [8]. To confirm the presence and examine the consequences of the A/G substitution in the four mouse strains at the mRNA level, genomic DNA as well as cDNA were sequenced and compared to that of C57BL/6J mice which demonstrate no evidence of mineralization. As shown previously, direct sequencing of exon 14 of the *Abcc6* gene in the four inbred strains confirmed the presence of a homozygous A nucleotide in position c.1863, while C57BL/6J harbors a G in that position [8], [9], [10], [11]. The A allele creates a new splice site resulting in a 5 bp deletion from the end of exon 14, a frame shift and a stop codon 200 nucleotides downstream from the site of the mutation. Thus, all four mouse strains with the A allele demonstrate similar consequences at the mRNA level.

**Figure 1:**
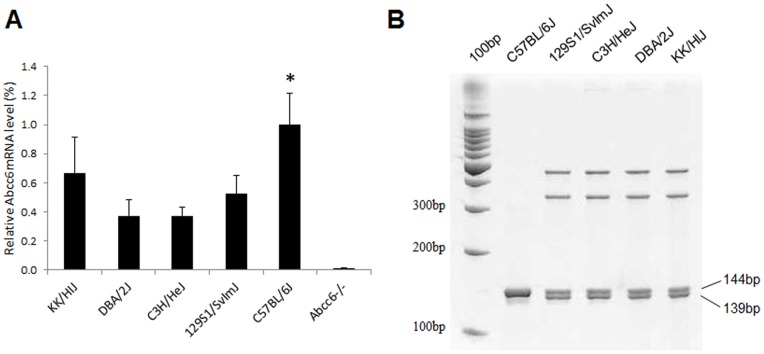
Analysis of *ABCC6* mRNA levels in the liver. (A) Relative mRNA levels in the mice with the A allele (KK/HlJ, DBA/2J, C3H/HeJ and 129S1/SvImJ), in comparison to the mouse with the G allele, C57BL/6J. Note that the mRNA level is essentially undetectable in *Abcc6^−/−^* mice; (n = 4–5; *p<0.05 in comparison to other groups; Student’s t-test). (B) Separation of the G and A alleles by fine resolution gel electrophoresis, represented by 144 and 139 bands, respectively.

To further examine the consequences of this mutation on *Abcc6* at the mRNA level, quantitative RT-PCR was performed. The *Abcc6^−/−^* mice had essentially undetectable levels of the corresponding mRNA, as compared to C57BL/6J mice (p<0.0015). The KK/H1J, DBA/2J, C3H/HeJ, and 129S1/SvImJ mice showed reduced, yet clearly detectable levels, in comparison to C57BL/6J mice (p<0.05), and Kruskal-Wallis test demonstrated significant difference overall (p<0.0083) (Fig. 1A). Fine resolution of the RT-PCR products on a polyacrylamide gel revealed the presence of the wild type allele as well as mRNA with the 5 base pair deletion (Fig. 1B) making these mice hypomorphic alleles. It should be noted that the shortened allele with a 5-bp deletion accounted for ∼60% of the total mRNA alleles.

**Figure 2:**
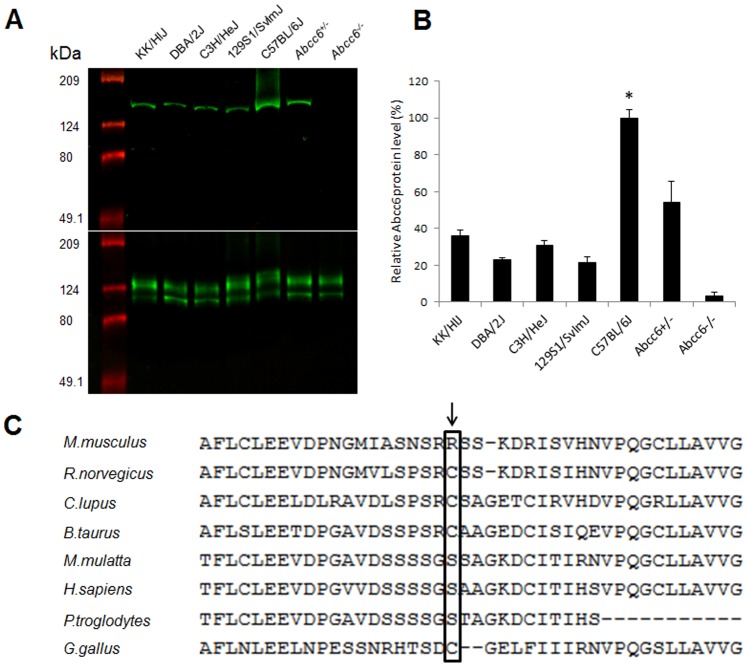
Western blot analysis of ABCC6 protein isolated from the liver of mice. (A) The blot was stained with a mouse anti-ABCC6 antibody S20 (upper panel). The same blot was rehybridized with an antibody for sodium potassium ATPase as a control of loading of plasma membrane proteins (lower panel). (B) The relative concentration of the protein, as determined from the Western blots in A by scanning densitometry, normalizing the ABCC6 protein levels by those with sodium potassium ATPase (n = 4–5; p<0.05 in comparison to other groups; Student’s t-test). (C) Lack of conservation of the arginine (R) in position p.621 of ABCC6 in different vertebrates.

**Figure 3:**
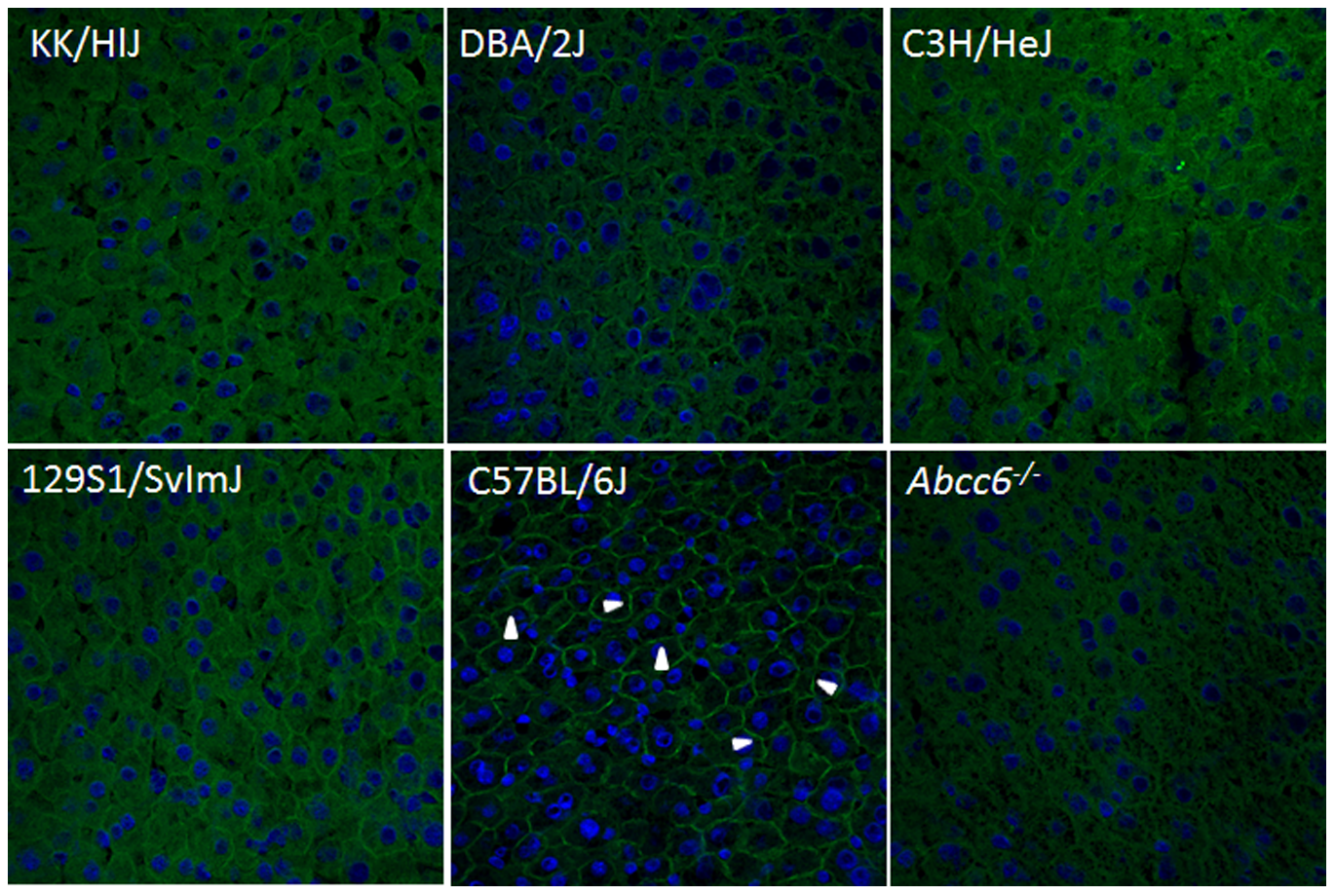
Immunofluorescence staining of Abcc6 in the liver of mice. Note positive staining associated with the plasma membranes in C57BL/6J mice (arrowheads) and complete absence of the staining in *Abcc6^−/−^* mice. A low, yet detectable level of immunofluorescence was noted in the four other strains which harbor the A allele of the *Abcc6* gene.

To examine the consequences of the *Abcc6* mutation at the protein level, Western blot analysis was performed with proteins extracted from the liver of these mice. While the *Abcc6^−/−^* mouse was completely devoid of ABCC6, the KK/HlJ, DBA/2J, C3H/HeJ, and 129S1/SvImJ mice demonstrated clearly detectable, yet markedly reduced levels of the ABCC6 protein in comparison to C57BL/6J (Fig. 2A). Quantitation of the protein by scanning densitometry, corrected for the level of sodium potassium ATPase as a marker of the plasma membrane proteins in the same protein samples, revealed that the expression of ABCC6 in the mutant mice was at ∼20–35% compared to C57BL/6J mouse (p<0.05) (Fig. 2B). The low level of protein expression was also demonstrated by semi-quantitative immunofluorescence of liver tissues from these mice. While C57BL/6J mice showed clearly detectable expression of the protein at plasma membranes of the hepatocytes, the *Abcc6^−/−^* mice showed complete absence of the protein. In contrast, KK/HlJ, DBA/2J, C3H/HeJ, and 129S1/SvImJ mice had a low, yet detectable level of expression of the protein (Fig. 3).

Since a significant amount of full-length ABCC6 protein was detected in the mice with the A allele, it was noted that the SNP, which was shown to result in aberrant splicing with frame shift of the *Abcc6* mRNA, could also result in a mutant, yet full-length protein with substitution of arginine by cysteine at position 621. However, phylogenetic examination of the amino acid in position 621 in the mouse Abcc6 revealed that this position is not conserved during vertebrate evolution (Fig. 2C). In fact, the amino acid at position 621 in rat, wolf, bovine, and chicken protein is cysteine, suggesting that the arginine-to-cysteine substitution in the mouse is not pathogenic. Consequently, this suggests that ∼20–35% of the Abcc6 protein is functional in these mice, yet they develop ectopic mineralization when stressed by dietary modifications (see below).

### Effect of Diet on Tissue Mineralization

**Figure 4:**
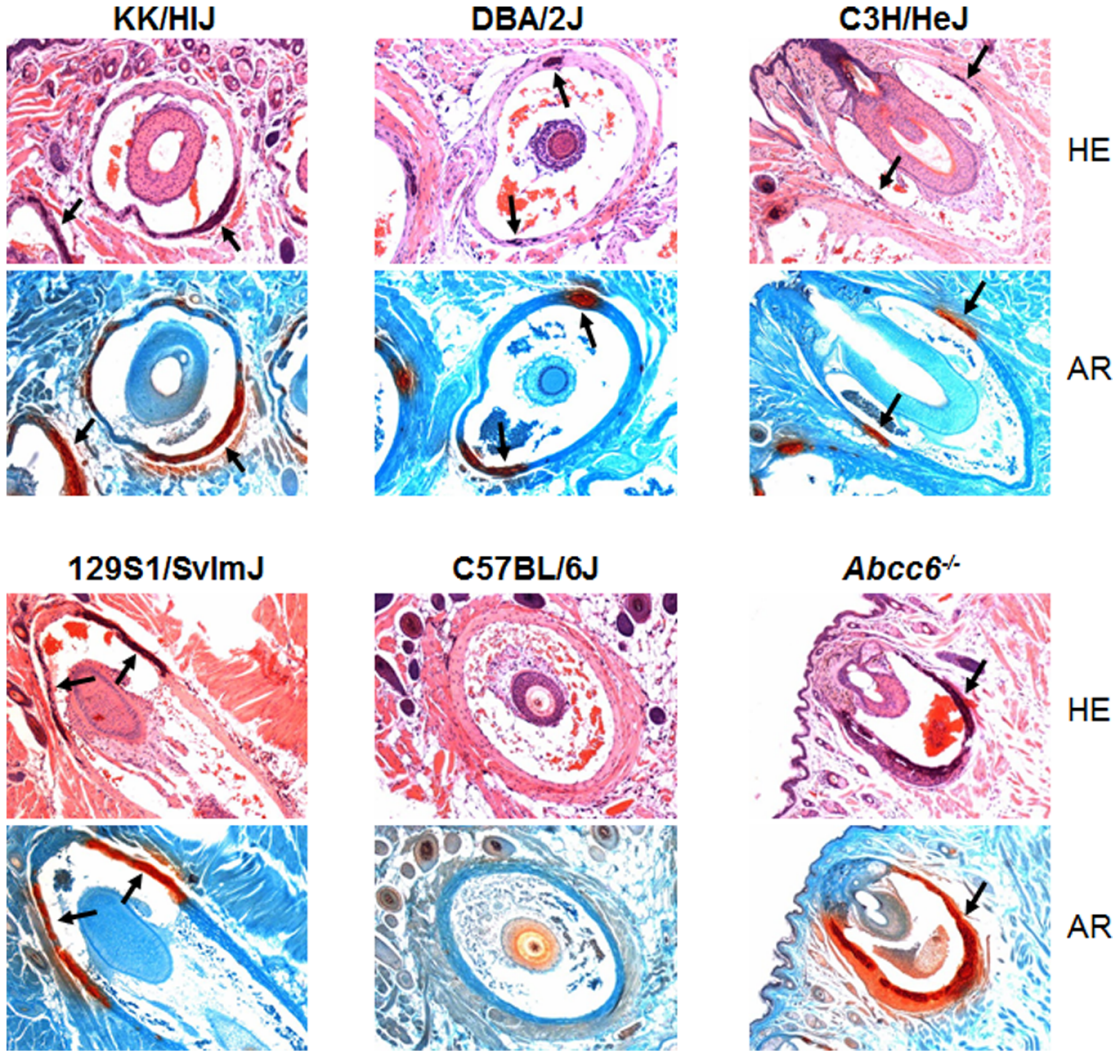
Mineralization of vibrissae dermal sheath in mice placed on experimental “acceleration diet” at the age of 4 weeks and sacrificed at 5 months of age. Note the absence of mineralization in the C57BL/6J mice and extensive mineralization in *Abcc6^−/−^* mice when examined by hematoxylin-eosin (HE) and by Alizarin red (AR) stains. Note ectopic mineralization in the four mouse strains with the A allele of the *Abcc6* gene (arrows).

We previously demonstrated that an experimental diet, so-called “acceleration diet”, can enhance the mineralization of the dermal sheath of vibrissae in *Abcc6^−/−^* mice; this diet consists of high phosphate and low magnesium [12]. To test whether this diet can also accelerate mineralization in C3H/HeJ, DBA/2J, and 129S1/SvImJ mice which show little, if any, mineralization when kept on standard rodent diet, the mice were placed on the acceleration diet at 4 weeks of age. At 5 months of age, muzzle skin containing the vibrissae was collected at the time of necropsy and stained for mineralization in the vibrissae dermal sheath by hematoxylin-eosin and Alizarin red stains. As expected, *Abcc6^−/−^* and KK/HlJ mice, which already showed mineralization when on standard rodent diet, now exhibited even more extensive mineralization (Fig. 4). In contrast, C57BL/6J mice showed no mineralization when kept on the same acceleration diet, while DBA/2J, C3H/HeJ, and 129S1/SvImJ now displayed extensive mineralization (Fig. 4; Table 1). These results indicate that the genetic background of different inbred mouse strains can modulate the ectopic mineralization process, and clearly, expression of the mineral deposition phenotype can be modulated by manipulating the mineral content in the diet.

## Discussion

Our studies demonstrate that inbred mouse strains with an allelic *Abcc6* mutation demonstrate ectopic connective tissue mineralization, as monitored by calcium deposition in the dermal sheath of vibrissae, yet the extent of mineralization varies under identical experimental conditions, including the same diet. The role of modifier genes in affecting the mineralization phenotype has been previously demonstrated by crossing *Abcc6^−/−^* mice with mice harboring mutations in the *Ggcx* gene encoding γ-glutamyl carboxylase, an enzyme critical for γ-carboxylation of matrix GLA protein, a potent anti-mineralization factor when in its fully carboxylated form [12], [14]. The *Abcc6^−/−^* and *Ggcx^+/−^* mice [5], [13] were generated on 129S1;B6 and 129S1;129×1;B6 mixed genetic backgrounds, respectively, and backcrossed with C57BL/6J for five generations; thus these strains differed by the 129×1 contribution to the background of the mice [12]. The results indicated that mineralization of the dermal sheath of vibrissae in *Abcc6^−/−^* mice takes place at ∼5–6 weeks of age and that mineralization is significantly enhanced at 3 months of age in comparison to wild type mice. However, the onset of mineralization in *Abcc6^−/−^*; *Ggcx^+/+^* mice was delayed until between 3–4 months of age, suggesting that the genetic background plays a role in modifying the mineralization process. In addition, the mineralization in the *Abcc6^−/−^*; *Ggcx^+/−^* was accelerated in comparison with the age-matched *Abcc6^−/−^*; *Ggcx^+/+^*, with ∼3-fold differences at 3, 4, and 9 months of age. These findings suggested a role for the *Ggcx* gene and the genetic background in modulating the phenotypic severity of mineralization in these mice [12].

PXE, while usually of late onset, shows complete penetrance, yet extensive phenotypic (both intra- and interfamilial) heterogeneity [1], [15]. Previous attempts to correlate the genotype of the patients with their phenotypic presentation have been unyielding, and no clear genotype/phenotype correlations could be established in different cohorts consisting of several hundreds of patients [15], [16]. It is also of interest to note that *ABCC6* mutations have been recently demonstrated in patients with generalized arterial calcification of infancy (GACI), a severe vascular mineralization disorder often diagnosed by prenatal ultrasound, with peri- and early postnatal demise in the majority of patients before 6 months of age [17], [18]. In some cases of GACI, the mutations in the *ABCC6* gene are the same as noted in the classic presentation of PXE with late onset of manifestations. Of interest is also one family where one of the siblings died from vascular calcification at early infancy while a brother developed late onset PXE shown to be associated with mutations in the *ABCC6* gene [19]. The exact reasons for this tremendous variability have not been disclosed, but there have been suggestions of genetic modifier genes and epigenetic influence, and various lifestyle variables, including diet, have been suggested to play a role in the phenotypic expression of PXE [3].

An interesting finding in our study was that inbred mouse strains, KK/HlJ, DBA/2J, C3H/HeJ and 129S1/SvImJ, showed ∼20–35% level of expression of ABCC6 at the protein level, as compared to C57BL/6J mice. Since PXE is an autosomal recessive disorder, and the heterozygous carriers of the mutations or the mice heterozygous for the SNP polymorphism do not show evidence of mineralization, these results suggest that the critical level for the expression of the mineralization phenotype is somewhere between 25% and 50% of the normal level of ABCC6. This observation is consistent with previous demonstrations that mice with β-thalassemia, which have ∼25% level of ABCC6 expression, do not develop mineralization phenotype [20]. These findings have implications for any potential therapy that is aimed to restore ABCC6 expression in the liver of patients with PXE, either through gene therapy, protein replacement, or cell-based therapy approaches [21]. At the same time, it is clear that the expression of Abcc6 in inbred mouse strains can result in varying degrees of mineralization dependent of the diet. Specifically, feeding the mice with “acceleration diet”, which has ∼2-fold elevated phosphate content and magnesium content reduced to 20% of the standard rodent diet, can significantly accelerate the mineralization process. Specifically, elevated magnesium content in the mouse diet, 5-fold higher than in the standard diet, can completely prevent the ectopic tissue mineralization in *Abcc6^−/−^* mice [22], [23]. It should be noted, however, that the high magnesium-containing diet can prevent the development of mineralization in *Abcc6^−/−^* mice but it does not reverse the existing mineral deposits [22], [23]. These observations would suggest that magnesium-containing diet might be effective in treatment of patients with PXE, but such treatment should be initiated as early as possible, as soon as the diagnosis has been established.

In summary, our studies on inbred mouse strains demonstrate considerable variability in ectopic mineralization of animals harboring the same mutation in the *Abcc6* gene, suggesting a role for genetic background genes. Furthermore, we have shown that the extent of mineralization and the onset of the mineralization process can be modulated by varying the mineral content of the diet. Finally, our results suggest that ABCC6 levels below ∼35% of the physiological levels may allow the ectopic mineralization processes to ensue. Collectively, the findings may have implications for development of therapies for PXE, a currently intractable disorder at the genome/environmental interface.
